# Productive Aging and Work: Overcoming Barriers to Creating Age-Friendly Workplaces Among Older Workers in Nigeria

**DOI:** 10.1093/geroni/igaa057.736

**Published:** 2020-12-16

**Authors:** Olubukola Omobowale

**Affiliations:** University of Ibadan, Nigeria, Ibadan, Oyo, Nigeria

## Abstract

Due to global demographic shifts, the issue of older workers’ health and productive aging is becoming much more pressing and Nigeria, the most populous country in Africa is not left behind. Productive aging involves providing a safe and healthy work environment for everyone using strategies that allow workers to function optimally at all ages, hence this study assessed the barriers to creating age friendly workplaces among older workers in Nigeria. A community based study was conducted among workers in Federal Government establishments aged 60 years and above. Using a Focus Group Discussion guide and an adapted checklist on age friendly workplaces, 16 strategic domains affecting older workers’ health and age friendly workplaces were explored. Data were analyzed thematically. A total of 150 older workers aged between 60-70 years were sampled. There is no formal older worker’s health initiative in all the establishments. Only two, of the sixteen domains checked were present. Other domains remain largely neglected by the management. Barriers to age-friendly workplaces include poor knowledge of the strategies, lack of political will and ageism. The concept of older workers’ health is a strange phenomenon among the respondents. Organizations need to invest in developing a robust package to help older workers use their skills and create age friendly workplaces that ensure safety, health and well-being of older workers, from their first day on the job to their last. Government policies targeted at productive aging and work should be put in place in order to create age-friendly workplaces for older workers.

